# Perceived Stigma Towards Psychological Illness in Relation to Psychological Distress Among Medical Students in Riyadh, Saudi Arabia

**DOI:** 10.1007/s40596-020-01247-4

**Published:** 2020-05-26

**Authors:** Dalal Ibrahim Alfayez, Norah Ali AlShehri

**Affiliations:** 1 King Saud University Medical City, Riyadh, Saudi Arabia; 2 King Saud University, Riyadh, Saudi Arabia

**Keywords:** Medical students, Psychological distress, Stigma

## Abstract

**Objective:**

Due to the high prevalence of psychological distress among medical students and its related functional and cognitive implications, this study aimed to investigate the association between perceived stigma and psychological distress, estimate the prevalence of each level of distress among medical students, and determine the independent significant risk factors of outcome variables for each level of psychological distress.

**Methods:**

A cross-sectional study was performed that surveyed medical students at King Saud University in 2018. Using the modified and validated stigma scale for receiving psychological help along with Kessler psychological distress scale, the survey measured perceived stigma towards mental illness in relation to the level of psychological distress.

**Results:**

Among the 524 participants, 395 surveys were completed. Participants had a mean age of 21.56 years old, and 53% were female. The overall prevalence of severe psychological distress was 30.7% (*N* = 161). Furthermore, 25.6% of participants reported experiencing moderate distress (*N* = 134). Additionally, a significant association was found between females and severe psychological distress. Moreover, family income was significantly associated with severe psychological distress in the extreme lower and upper groups (5000–10,000 SR and above 20,000 SR). Participants with high levels of psychological distress were more likely than those with low levels to agree or strongly agree with 3 out of 10 items related to perceived stigma.

**Conclusions:**

Medical students with moderate/severe psychological distress disclosed more concerns regarding stigma, particularly about perceived consequences of their mental health issues being revealed to others. Such opinions could cause physical health problems and decrease quality of life.

Psychological distress poses a substantial public health concern that affects people all around the world. The term psychological distress has been defined as a state of emotional suffering characterized by symptoms of depression and anxiety that are sometimes accompanied by somatic symptoms (e.g., insomnia, headaches, lack of energy) [1, 2]. Such suffering can have a tremendous effect on an individual, both directly and indirectly. Psychological distress has been shown to cause cognitive impairment and physical health issues such as arthritis, cardiovascular disease, and chronic obstructive pulmonary disease [3, 4].

Medical students in particular were found to experience great levels of stress during their academic years [5, 6]. Such distress may influence their concentration, learning, and ability to memorize information well, which subsequently affects their educational achievement and later careers.

Due to the wide range of scales used to assess psychological distress, no single value can be specified regarding its global prevalence; however, its prevalence approximately ranges between 5% and 27% in the general population [7–9]. Furthermore, it has been observed that medical students also suffer from high levels of psychological distress, regardless of their medical knowledge. For example, in 2017, Al Saadi showed that 52.6% of Syrian medical students at Damascus University suffered from psychological distress [10]. Additionally, in 2007, Dyrbye found that 49% of 1701 medical students from five different universities in the USA had depressive symptoms [11].

In Saudi Arabia, epidemiological data on psychological disorders among the general population are scarce. However, studies have been conducted on psychological distress in particular populations and age groups. Multiple local studies have been conducted to estimate the prevalence of psychological distress and depressive symptoms among medical students [12, 13]. About 64% of King Saud University medical students reported having psychological distress during the 2007–2008 academic year [12]. In addition, 46.2 % of King Saud University medical students reported having depressive symptoms in 2012–2013 [13].

In most studies, two characteristics were found to be worthy of attention: gender and age; the prevalence of psychological distress has been found to be higher among females of all age groups [14–17] than males in the USA, Australia, Syria, and Saudi Arabia [1, 18, 19]. Furthermore, it was previously observed that the prevalence of psychological distress tends to decrease in late adolescence and early adulthood age groups [1, 8, 18, 20].

Due to the alarmingly high prevalence of psychological distress and depressive symptoms among medical students [12, 13], early recognition and treatment are important to provide effective management and avoid adverse negative outcomes. However, none of these local studies addressed the effects of perceived stigma on mental illnesses, which may lead to self-neglect and subsequent underdiagnoses and lack of treatment, particularly among medical students.

Additionally, one of the major barriers to self-awareness regarding psychological disorders is perceived stigma by either the public or the patients themselves. Stigma has been described as “a cluster of negative attitudes and beliefs that motivate the general public to fear, reject, avoid, and discriminate against people with mental disorders” [21]. Considerable researches affirm stigma to be recognized as a barrier to recovery; stereotypes, prejudice, and discrimination against mentally ill patients will affect person’s sense of self and consequently withhold seeking for help and adhering to ongoing treatment [22].

The objectives of the current study were as follows: to examine the association between perceived stigma and psychological distress, estimate the prevalence of each level of psychological distress among medical students, and determine the independent significant risk factors for all outcome variables (e.g., GPA, year of study, gender, and age) of each level of psychological distress.

## Methods

This study used a cross-sectional design to explore perceived stigma in relation to the level of psychological distress among medical students, conducted in 2018–2019. The institutional review board at King Saud University approved the study prior to the recruitment of participants. All participants were current medical students at King Saud University from all five academic years. Students were invited via e-mail through the student council to participate in the study by voluntary completing an online survey. Out of 1500 medical students, 525 agreed to participate in the study (35%) and 395 completed the survey (26.3%); responses were anonymized by using electronic survey with non-identifying questions. Completed questionnaires were collected 2 months before the university’s examination period so that actual examination stress would not affect the students’ responses.

### Study Measures

The survey included sociodemographic items (age, gender, academic year, GPA, marital status, and family income), standard instruments to measure dimensions of psychological distress (Kessler psychological distress scale) [23], and items exploring perceived stigma regarding mental illness [24].

#### Psychological Distress

The six-item Kessler psychological distress scale (K6) is the most widely used screening scale of non-specific psychological distress providing an estimate prediction of the prevalence of serious mental illness, defined as meeting diagnostic criteria for a DSM-IV disorder in the past 12 months and experiencing significant impairment with sensitivity in the upper 90th–99th percentile range [25]. The validity of this scale is well-established internationally including in populations of medical students, higher K6 scores were reported by individuals receiving one or more mental health treatment [26]. Frequency of feeling nervous, hopeless, restless, or fidgety, so sad that nothing could cheer them up and that everything was an effort and worthless, were assessed and graded. Each item is scaled from zero to four, with zero meaning “none of the time” and four meaning “all of the time.” Total scores range between 0 and 24, and are further divided into three categories: scores between 13 and 24 represent high psychological distress; scores between 8 and 12 indicate moderate psychological distress; and scores between 0 and 7 represent low psychological distress [23]. Participants who had high scores representing moderate to severe psychological distress could not be reached individually due to the anonymous nature of the survey; therefore, an official e-mail was sent through the student council to all students thanking them for their participation along with providing guidance for accessing school-specific mental health resources.

#### Stigma

Stigma involves multiple dimensions, including social stigma and self-stigma. Validated scales measuring stigma for receiving professional psychological help assessing these dimensions [22, 27] were previously selected and modified by other researches to be more relevant to medical students [24]. Liselotte N. Dyrbye, the author of “The Impact of Stigma and Personal Experiences on the Help-Seeking Behaviors of Medical Students with Burnout,” was contacted via e-mail and granted her permission to use and print the survey [24]. The questionnaire consists of 10 questions regarding the dimensions of stigma. Self-stigma refers to medical students thinking it is a sign of personal weakness or inadequacy to receive treatment for emotional or mental health problems. Social stigma focuses on the individuals around medical students, including residency directors, supervisors (e.g., faculty, residents, deans), fellow students, and patients, and how these individuals would view the students if they were aware they had received treatment for an emotional or mental health problem. Additionally, the questionnaire includes questions regarding the confidentiality of medical students, if they were to receive mental health treatment.

### Analysis

Based on previous literature, the use of the most conservative choice with *p* = 0.5. To ensure the estimation error would be at most alpha 0.05 with 95% confidence, a sample size of 416 was required with assumption of 10% non-response. Categorical data were summarized with absolute numbers and percentages. Numeric data were summarized with mean and standard deviation (SD). Comparisons between different groups were performed using Chi-square test or Fisher exact test for categorical variables. Multiple logistic regression models were used to identify independent risk factors for stress. To quantify multivariate association, odds ratio with 95% confidence intervals (CI) was estimated. All the analysis was performed using the [SAS/STAT] software, version [9.2] (SAS Institute Inc., Cary, NC, USA.). A 2-sided *p* value < 0.05 was considered statistically significant.

## Results

### Sociodemographic Data

Out of 1500 medical students, 524 responded to the online survey. From that group, 395 surveys were completed, for a completion rate of 26.3%. Table 1 describes demographic characteristics of the participants. Participants had a mean age of 21.56 (SD 1.68), and more than half were female (253; 53%). The majority were in their fourth year of medical school (110; 23.3%), single (455; 96%), and had a high family income exceeding 20,000 Saudi Riyals (271; 57.4%). More than half of the students had good cumulative grade point averages, ranging from 4–4.74 out of 5 (252; 54.8%).

**Table 1:** Characteristics of study subjects

Variable	Level	*N* = 524	Percent
Age	Mean	21.56	-
	SD	1.68	-
Gender	Male	219.00	46.4
	Female	253.00	53.6
Year	1st	82.00	17.4
	2nd	93.00	19.7
	3rd	101.00	21.4
	4th	110.00	23.3
	5th	86.00	18.2
Marital status	Single	455.00	96.4
	Married	13.00	2.8
	Divorced	4.00	0.8
Family income	< 5000 SR	22.00	4.7
	5000–10000 SR	46.00	9.7
	> 10000–20000 SR	133.00	28.2
	> 20000	271.00	57.4
GPA	4.75–5	114.00	24.8
	4–4.74	252.00	54.8
	≤ 3.99	94.00	20.4

### Psychological Distress Prevalence

The overall prevalence of severe psychological distress was 30.7% (*N* = 161). Additionally, 25.6% of participants reported having moderate distress (*N* = 134), providing an overall prevalence of moderate to severe psychological distress of 56.3% (*N* = 295).

In a review of the associations between demographic characteristics and level of psychological distress (see Table 2), a significant association was found between females and severe psychological distress (*p* < 0.001). Moreover, family income was significantly associated with severe psychological distress in the extreme lower and upper groups (5000–10,000 SR and above 20,000 SR; *p* = 0.013 and 0.016, respectively).

**Table 2:** Multivariate analysis of the association between demographic characteristics and level of psychological distress

		Response severe stress	
Covariate	Level	Multivariate OR (95% CI)	*p* value
Gender	Female	1.67 (1.339, 2.09)	*< 0.001*
Year	2nd	0.83 (0.516, 1.34)	0.452
	3rd	1.00 (0.673, 1.47)	0.982
	4th	1.03 (0.646, 1.63)	0.911
	5th	1.42 (0.708, 2.83)	0.325
Marital status	Married	1.58 (0.525, 4.78)	0.415
	Single	0.77 (0.343, 1.74)	0.532
Family income	5000–10,000 SR	2.00 (1.158, 3.45)	*0.013*
	> 10,000–20,000 SR	0.86 (0.568, 1.32)	0.498
	> 20,000	0.63 (0.434, 0.92)	*0.016*
GPAC	4–4.74	0.88 (0.665, 1.17)	0.377
	4.75–5	0.87 (0.605, 1.24)	0.437
Age	1 year increase	0.91 (0.703, 1.17)	0.455

The italicized values demonstrate statistical significance considering a 2 sided *p* value < 0.05

### Perceived Stigma Towards Psychological Health Problems, Fear of Discrimination, and Privacy Breaches

Participants’ perceptions of self-stigma were relatively low, and 70.2% (277 of 524) of participants disagreed or strongly disagreed that it is a sign of personal weakness or inadequacy to receive treatment for emotional or mental health problems. However, when it comes to social stigma, around half of study subjects agreed or strongly agreed that residency directors, supervisors, fellow students, and patients would negatively react if they were aware the student had or was on treatment for emotional or mental health problem (161; 40.7%, 218; 55.2%, 171; 43.3%, 2663; 66.6%). Participants, however, were not sure about confidentiality regarding emotional or mental health problems. A total of 50.4%, neither agreed nor disagreed that mental health care provided to medical students by their school/affiliated institution is truly confidential (199 of 524), or that deans (44.3%;175) or residency program directors (41%; 162) could access their personal medical records if they wished to do so. However, 45% (178 of 524) disagreed or strongly disagreed that if they sought treatment for an emotional or mental health problem, it might end up in their academic record. A similarly high percentage (61.8%; 244) agreed or strongly agreed that if they were to receive treatment for an emotional or mental health problem, they would hide it from people.

All ten items of stigma showed a statistically significant association with participants having moderate to severe psychological distress; therefore, a multiple logistic regression model was used to identify independent variables (see Table 3). Table 3 shows that in comparison with respondents with mild psychological distress, those with moderate to severe psychological distress were more likely to perceive stigma. For instance, participants with higher level of psychological distress were more likely to agree or strongly agree that it is a sign of personal weakness or inadequacy to receive treatment for emotional or mental health problems (OR = 1.80; *p* = 0.003). In addition, they agreed or strongly agreed that fellow students would see them in a less favorable way if they found out the participant had received treatment for emotional or mental health problems (OR = 1.36; *p* = 0.015). Furthermore, they disagreed or strongly disagreed that mental health care provided by their school/affiliated institution is truly confidential (OR = 1.46; *p* = 0.012).

**Table 3:** The association between stigma and moderate to severe psychological distress using multiple logistic regression model OR, CI, and *p* value

		Response = severe + moderate stress	
Covariate	Level	Multivariate OR (95% CI)	*p* value
Stigma1	Strongly agree + agree	1.80 (1.221, 2.66)	*0.003*
Stigma2	Strongly agree + agree	0.97 (0.758, 1.25)	0.824
Stigma3	Strongly agree + agree	1.02 (0.789, 1.32)	0.868
Stigma4	Strongly agree + agree	1.36 (1.061, 1.75)	*0.015*
Stigma5	Strongly agree + agree	1.05 (0.831, 1.33)	0.673
Stigma6	Strongly disagree + disagree	1.46 (1.089, 1.97)	*0.012*
Stigma7	Strongly agree + agree	1.08 (0.798, 1.45)	0.631
Stigma8	Strongly agree + agree	1.20 (0.893, 1.61)	0.228
Stigma9	Strongly agree + agree	1.23 (0.925, 1.64)	0.155
Stigma10	Strongly agree + agree	1.23 (0.986, 1.53)	0.067

The italicized values demonstrate statistical significance considering a 2 sided *p* value < 0.05

## Discussion

Mental and emotional disorders are known causes of reduced work and school performance; therefore, it is important that mental health issues are identified and treated early. In this study, the K6 was used to assess the emotional and mental health of medical students [23]. The overall prevalence of moderate to severe psychological distress was 56.3%, which showed that the medical students who participated in our study experienced relatively high levels of psychological distress. Abdulghani et al. showed a similarly high prevalence of stress among medical students at King Saud University in 2011, reaching 63.8% [12]. This may be attributed to the similarity in the scale (Kessler 10) used in their study. A previous study performed by Al Saadi in 2017 among Syrian medical students at Damascus University showed that psychological distress was estimated to be 52.6% in that population [10]. In addition, a recent meta-analysis reviewed 77 studies, included in which were data from 62,728 medical students, and found the global prevalence of depression to be 28% [28]. Such a high prevalence of emotional disorders might be attributed to the stigmatization of mental illness and the fear of being exposed experienced by individuals with mental health disorders.

Our findings showed that females were more affected than males, which offered some confirmation for the previous analysis by Abdulghani measuring stress and its effects on 775 medical students at King Saud University [12]. Furthermore, Al Saadi showed that females were twice as likely to show symptoms of depression and anxiety as males in a population of Damascus University Syrian medical students [10].

In the current study, family income was identified as a risk factor for psychological distress. Medical students with low (5000–10,000 SR) or high family incomes (> 20,000 SR) had a significant association with severe psychological distress. This factor was not either addressed in similar studies, or was considered, but did not show any significance [10, 12, 24]. For example, Al Saadi reported that personal income was more significantly related to psychological distress than family income among Syrian medical students [10].

In contrast to previously published studies [12, 13, 24], the current study showed no significant difference in distress levels among different stages of medical education. This was not in line with other studies that suggested different findings, and it was reported in Abdulghani study that levels of distress were highest among first-year students (78.7%) in Saudi Arabia [12]. Additionally, in 2005, Dahlin studied 342 medical students at Karolinska Institute, Sweden, in their first, third and sixth years. Higher level of stress was observed in first year students compared to third and sixth. [29]. On the contrary, Shaikh and Saipanish reported higher level of stress among third- and fourth-year medical school students in Pakistan and Thailand. Such discrepancy could be attributed to the different scales used to measure distress in these studies [30, 31].

As for marital status, no significant association was observed in this study, which was anticipated, given the low percentage of participants who were married (2.8%), compared with those who were single which is in line with the overall school demographics [32]. In contrast, a systematic review performed by Dyrbye in 2006 indicated that multiple studies showed lower stress rates among married students, relative to their single counterparts [28].

This questionnaire used in this study provided a description of the beliefs about stigma perceived by medical students reporting psychological distress, as well as whether these beliefs regarding emotional or mental health problems were affected by their current distress levels. The findings of this study indicated that perceived stigma can be distorted by current states of emotional or mental health. For example, participants with severe psychological distress had higher scores related to stigma, compared with those with a lower level of distress. This is in line with the research findings of Dyrbye and colleagues [24]. Perceived stigma and fears of being judged and privacy breaches were shown to a great extent in participants with moderate to severe psychological distress, unlike those with low psychological distress. This finding corresponds to that of Dyrbye and colleagues’ multi-institutional study, in which medical students with burnout more frequently expressed beliefs in mental illness stigmatization [24].

Medical school is a competitive environment for students, in which they try to be the best version of themselves. This could explain why a fair amount of those students who undergo psychological distress feel weak when seeking treatment. In comparison with participants with mild psychological distress, those with moderate to severe psychological distress were more likely to agree or strongly agree that it is a sign of personal weakness or inadequacy to receive treatment for emotional or mental health problems. These findings are similar to those from a study by Schwenk, in which most students with moderate to severe depression agreed that if they asked for help, they would be admitting to themselves that their coping skills are inadequate, or would be blamed by others [33].

A previous study reported that in two medical schools in the UK, fear of being stigmatized was one of the barriers preventing medical students from seeking help [34]. The high percentage of medical students who agreed that program directors, supervisors, fellow students, and even patients would change their attitude and thoughts towards them if they knew about their mental health issues may have created a sense of insecurity and increased their efforts to conceal whatever distress they were experiencing. In the current study, most participants preferred to hide their emotional and mental health problems and were not quite confident regarding the confidentiality of their medical records. In another cross-sectional study, 16% of students observed supervisors violating the privacy of personal information of other students with emotional issues, making it an actual reality, rather than a fear [24].

This study had some limitations. One limitation was the use of a self-report survey, which may have created a possible recall bias. In addition, due to the study design, causal relationships could not be investigated. Furthermore, although medical students in this study were from a nearly similar sample in terms of age and profession, other known medical and psychological problems which can create great psychological distress were not addressed in the survey.

In conclusion, medical students with moderate to severe psychological distress (56.3%), who were mostly females (65.22%), disclosed more concerns regarding mental health stigma, affirming that it is a sign of personal inadequacy to receive treatment for a psychological health problem, doubting the confidentiality of mental health care provided by their institution and fearing that fellow students would condescend if they found out they had received psychological treatment. Such opinions could cause emotional and cognitive impairment and physical health problems, and ultimately reduce the quality of care provided to patients if distress was unrecognized and untreated for several years. The study findings implicate the importance of educational emphasis on mental disorders as a physical illness rather than a mental weakness, in addition to the seriousness of patient confidentiality and its subsequent effect on psychologic distress level. Further research is needed to look into the actions causing these confidentiality concerns and fear of stigmatization to benefit future physicians and their patients.
